# A Novel Nitrogen-Fixing Bacterium *Raoultella electrica* Isolated from the Midgut of the Leafhopper *Recilia dorsalis*

**DOI:** 10.3390/insects14050431

**Published:** 2023-04-30

**Authors:** Qiuyan Huang, Yilu Feng, Hong-Wei Shan, Jian-Ping Chen, Wei Wu

**Affiliations:** State Key Laboratory for Managing Biotic and Chemical Threats to the Quality and Safety of Agro-Products, Key Laboratory of Biotechnology in Plant Protection of Ministry of Agriculture and Zhejiang Province, Institute of Plant Virology, Ningbo University, Ningbo 315211, China; nbuhqy@foxmail.com (Q.H.);

**Keywords:** leafhopper, nitrogen fixation, *Recilia dorsalis*, symbiotic microorganisms, *Raoultella*, genome

## Abstract

**Simple Summary:**

Nitrogen is a vital element that all living organisms require for growth and development. Although the atmosphere has an abundant supply of gaseous nitrogen, unfortunately, most organisms are unable to utilize it. Some microorganisms are capable of converting nitrogen gas to ammonia, which serves as a nitrogen source available to most organisms. Ammonia is subsequently utilized by organisms to synthesize a range of nitrogen-containing compounds that are necessary for life activities. For example, nitrogen-fixing microorganisms aid leguminous plants in obtaining the required nitrogen. Herbivorous insects face significant growth and development restrictions due to low total nitrogen content in their food. In this study, we isolated a nitrogen-fixing bacterium, *Raoultella electrica*, from the digestive tract of the leafhopper, *Recilia dorsalis*. *R. electrica* has all the nitrogen fixation genes and colonizes the gut lumen of leafhoppers. We further evaluated the growth rate of *R. electrica* in nitrogen-containing and nitrogen-free media and measured its nitrogenase activity through an acetylene reduction assay. These findings may be useful in studying the biological nitrogen fixation by gut microbes in host insects.

**Abstract:**

Nitrogen is a crucial element for the growth and development of insects, but herbivorous insects often suffer from nitrogen nutrition deficiencies in their diets. Some symbiotic microorganisms can provide insect hosts with nitrogen nutrition through nitrogen fixation. Extensive research has clearly demonstrated the process of nitrogen fixation by symbiotic microorganisms in termites, while evidence supporting the occurrence and significance of nitrogen fixation in the diets of the Hemiptera is less conclusive. In this study, we isolated a strain of *R. electrica* from the digestive tract of a leafhopper, *R. dorsalis*, and found that it had nitrogen-fixing capabilities. Fluorescence in situ hybridization results showed that it was located in the gut of the leafhopper. Genome sequencing revealed that *R. electrica* possessed all the genes required for nitrogen fixation. We further evaluated the growth rate of *R. electrica* in nitrogen-containing and nitrogen-free media and measured its nitrogenase activity through an acetylene reduction assay. The findings of these studies could shed light on how gut microbes contribute to our understanding of nitrogen fixation.

## 1. Introduction

Many herbivorous insects typically have an imbalanced diet, often feeding on foods with high C:N ratios, such as bark, leaves, or sap, that lack certain amino acids and have limited nitrogenous nutrients [1,2]. Insect symbiotic microorganisms play a crucial role in providing herbivorous insects with the necessary nitrogen nutrients [3], usually by enriching nitrogen and recycling nitrogen waste [4,5]. Additionally, a few species of symbiotic microorganisms can convert nitrogen into nitrogenous organic compounds available to insects through nitrogen fixation [6]. Symbiotic nitrogen fixation was initially observed in termites [7,8], but further studies have proven the existence of nitrogen-fixing microbes in the digestive systems of several insect species, including Coleoptera, Diptera, and Hemiptera [6,9,10,11]. These nitrogen-fixing bacteria in insects mainly belong to the phyla Proteobacteria, Firmicutes, Actinobacteria, and Cyanobacteria [6,9,10,11].

The *Raoultella* genus is a gram-negative, aerobic, non-motile rod that belongs to the Enterobacteriaceae family in the phylum Proteobacteria. The genus *Raoultella* was previously classified under the *Klebsiella* genus. In 2001, the classification of the *Klebsiella* genus was revised to include the *Raoultella* genus as a separate classification based on the results of a phylogenetic analysis that examined 16S rRNA and rpoB, along with specific biochemical activities [12]. There are four known species of *Raoultella*: *Raoultella ornithinolytica*, *Raoultella planticola*, *Raoultella terrigena*, and *Raoultella electrica* [13,14,15,16]. These bacteria have been found in various environments, such as water, soil, insects, fish, ticks, and termites [17,18,19,20,21]. Recent studies have revealed the nitrogen fixation capacity of several *Raoultella* species, including *R. terrigena* R1Gly extracted from tobacco, *R. terrigena* DR-E5 isolated from the gut of the bark beetle *Dendroctonus valens*, and *R. ornithinolytica* BAL286 isolated from temperate estuarine surface waters [17,18,19].

The leafhopper *Recilia dorsalis* (Cicadellidae: Deltocephalinae) is a notorious pest of rice and is widely distributed among rice-producing areas in Asia. It can transmit various rice viral pathogens, such as Rice dwarf virus, Rice stripe mosaic virus, and Rice tungro virus, through continuous reproduction during the sap-sucking process [22,23]. Leafhoppers are associated with the obligate bacterium “*Candidatus* Sulcia muelleri” and a partner β-proteobacterium, which provides them with 10 essential amino acids (EAAs) and other nutrients that are lacking in their diet [24]. While various symbiotic bacteria have been found in leafhoppers that provide fitness benefits to the insect host, such as growth rate, size, and reproductive ability [25,26,27,28,29], none have been shown to have the ability to fix nitrogen. In this study, we isolated a strain of *R. electrica* Rd210413 from the digestive tract of leafhoppers *R. dorsalis*, which was found to possess nitrogen fixation capacity. We analyzed the complete genome sequence and characteristics of the strain and evaluated its nitrogen fixation capacity using an acetylene reduction assay.

## 2. Materials and Methods

### 2.1. Isolation of the Bacterial Strain and Culture Condition

**Figure 1:**
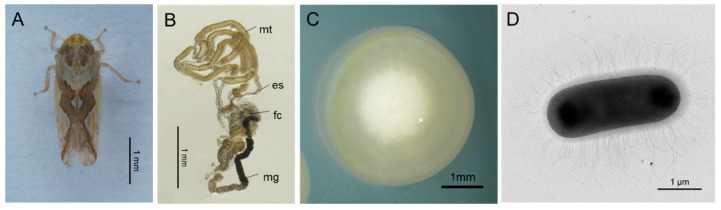
Isolation of *R. electrica* from the digestive tract of the leafhopper *R. dorsalis*. (**A**) Male adult of the leafhopper *R. dorsalis*. Bar, 1 mm. (**B**) Digestive tract of *R. dorsalis*. Bar, 1 mm. (**C**, **D**) The colony morphology (**C**) and cell morphology (**D**) of *R. electrica* on LB medium plate after 3 days of growth. es, esophagus; fc, filter chamber; gl, gut lumen; me, midgut epithelium; mg, midgut; mt, Malpighian tubules.

The leafhopper *R. dorsalis* adults were collected from a rice field in Jiaxing, Zhejiang Province, China, and maintained in insect-proof greenhouses for 2 years at 26 ± 1 °C, with a 16:8 h light-to-dark cycle and 50 ± 5% relative humidity on the rice variety Taichung Native 1 (TN1). We obtained fifty adult *R. dorsalis* and subjected them to surface sterilization using 70% ethanol, followed by washing with ddH_2_O. The whole digestive tracts of the *R. dorsalis* were carefully dissected under sterile conditions in ice-cold sterile phosphate buffer solution (PBS, pH = 7.2) (Figure 1A,B). The digestive tracts were ground in 100 μL PBS, diluted 1000 times, and applied onto LB agar plates. These plates were then incubated under both aerobic and anaerobic conditions (using AnaeroGen; Oxoid, Basingstoke, United Kingdom) for 48 h at 28 °C. Pure colonies were obtained after selecting colonies with different morphological characteristics based on color, shape, size, and rough or smooth surface and rescored on fresh LB medium at least 3 times. In order to screen for enteric bacteria with nitrogen fixation ability, the pure culture bacterial strains were transferred to Ashby plates for further culture. Eventually, a strain of gut bacteria with nitrogen fixation ability was obtained through this process.

### 2.2. Bacterial Identification and Phylogenetic Analysis

The universal bacterial primer pairs 27F (5′-AGAGTTTGATCMTGGCTCAG-3′) and 1492R (5′-TACGGYTACCTTGTTACGACTT-3′) were used to amplify the 16S rRNA gene for the identification of bacterial strains. To perform phylogenetic analysis, the obtained 16S rRNA gene sequence was analyzed using the BLAST program available on GenBank (https://www.ncbi.nlm.nih.gov/) (accessed on 21 February 2023) and EzBioCloud (http://www.ezbiocloud.net/) (accessed on 21 February 2023). The obtained sequence data were aligned using the CLUSTAL W program with gap deletion. The phylogenetic tree was constructed using MEGA 7 with the Maximum Likelihood method based on the Hasegawa–Kishino–Yano model, and bootstrap analysis was performed with 1000 replications to determine the reliability of individual branches in the phylogeny.

### 2.3. De Novo Sequencing, Genome Assembly and Annotation

The desired genomic DNA of *R. electrica* strain Rd210413 was extracted using the Ezup column-type bacterial genomic DNA extraction kit (Sanggong, Shanghai, China). The genome was de novo sequenced using a combination of high-throughput Illumina HiseqX-Ten and PacBio RSII long-read sequencing platforms at Shanghai OE Biotech Co., Ltd. A DNA library with 300 bp inserts was constructed and sequenced using Illumina HiseqX-Ten, while a 10 kb library was generated and sequenced using the PacBio RSII platform. The raw reads were initially assembled using Falcon, and the resulting consensus sequences were circularized using Circulator, resulting in a closed genome sequence. Gene prediction for the assembled genome of *R. electrica* utilized prodigal, RNAmmer, RNAscan-SE, Rfam, and RepeatMasker software. The predicted amino acid sequences were compared to databases including COG, GO, KEGG, NR, and Swiss-Prot using NCBI BLAST. Annotation of genes and their functions were combined to generate the annotation for *R. electrica*. The Circos software was used to display the genome and analyze noncoding RNA and gene function annotations, constructing a genome-wide map of the strain.

### 2.4. Fluorescence In Situ Hybridization (FISH)

A total of thirty leafhoppers were collected, extracted their intact digestive tracts through dissection and subsequently fixed them in 4% paraformaldehyde at 4 °C overnight. After fixation, the tissues were pretreated in hybridization buffer (20 mM Tris-HCl, 180 mM NaCl, 10% *v*/*v* SDS, 30% *v*/*v* formamide) for 15 min and then incubated in hybridization buffer containing 10 nM oligonucleotide DNA probes targeting the 16S rRNA sequence of *R. electrica* (5′-GCGGTGAGGTTAATAACCTCATCG-3′-Cy3). Following a 4-h incubation at 50 °C, the samples were thoroughly washed with washing buffer (0.15 M NaCl, 0.015 M sodium citrate) and then observed using a Leica TCS SP8 confocal microscope.

### 2.5. Transmission Electron Microscopy

To analyze the morphology of *R. electrica*, specimens were subjected to transmission electron microscopy using a conventional negative staining procedure. The *R. electrica* were adsorbed onto copper grids coated with formvar film, fixed with 2.5% glutaraldehyde for 1 min, and stained with 2% (*w*/*v*) sodium phosphotungstate for 1 min. The stained specimens were observed using a Hitachi electron microscope HT7800.

### 2.6. Nitrogen Fixation Capacity

To test the nitrogen-fixing ability of *R. electrica* strain Rd210413, we first analyzed its growth on LB and Ashby nitrogen-free medium and *Bradyrhizobium japonicum* and *Escherichia coli DH5α* as positive and negative controls, respectively. The bacteria were grown in LB liquid medium until OD600 reached 0.6, then collected by centrifugation. For Ashby nitrogen-free medium, cells were washed three times with a sterile Ashby nitrogen-free liquid medium and incubated at 28 °C for 12 h to deplete nutrients. Prior to the experiment, bacteria were adjusted OD600 to 0.1. For spot dilution growth analyses, the bacterial liquid was serially diluted (1:10), and 2 μL was spotted on LB or Ashby nitrogen-free medium at 28 °C. Growth curves of bacteria in LB or Ashby nitrogen-free liquid media were recorded using Bioscreen C at 28 °C, and OD 600 was measured every 30 min until all strains reached the stationary phase.

Subsequently, the *R. electrica* strain Rd210413 was tested for bacterial nitrogenase activity using the acetylene reduction assay. The cells were grown in LB liquid medium until reaching the exponential phase, harvested by centrifugation, washed with sterilized Ashby nitrogen-free liquid medium, and resuspended in 20 mL of sterilized Ashby nitrogen-free liquid medium in a sealed 60 mL bottle with He:C_2_H_2_ (90:10, *v*/*v*) gas mixture. After 24 h of cultivation at 28 °C, 1.0 mL of mixed gas from each bottle was extracted using a microsyringe and analyzed for ethylene concentration using gas chromatography (GC-2014, Shimadzu) equipped with a flame-ionization detector and GDX-502 packed column. The GC operating conditions were as follows: column temperature set to 65 °C, and detection and vaporization chamber temperatures set to 110 °C. Nitrogen gas was used as the carrier with a flow rate of 35 mL/min. Gas (H_2_) pressure and air pressure were maintained at 0.8 kg/cm^2^ and 1.6 kg/cm^2^, respectively. A negative control using pure He gas was also conducted.

To investigate the expression of nitrogen fixation genes in *R. electrica* under different nitrogen conditions, the nifH gene expression was measured by RT-qPCR in *R. electrica* grown on LB and Ashby media for 48 h. RNA isolation was performed using RNAiso reagent (TaKaRa, China) and cDNA synthesis was carried out using the HiScript^®^II Q Select RT SuperMix for qPCR (+ gDNA wiper) Kit (Vazyme, Nanjing, China). qPCR was conducted using the QuantStudio 5 Real-Time PCR System (ThermoFisher, USA) and 2×ChamQ SYBR qPCR Master Mix (Vazyme, Nanjing, China). The ΔΔCT method was used to analyze nifH expression levels, with 16S rRNA as the internal control. The nifH forward primer sequence was 5′-AGGCGGTATCGGCAAATC -3′, and the reverse primer sequence was 5′-ATCATCACTTTCTTACCCATCTCC-3′. The 16S rRNA forward primer sequence was 5′- GTGCCCTTGAGGCGTGGCTT-3′, and the reverse primer sequence was 5′-TTGCGCTCGTTGCGGGACTT -3′.

## 3. Results

### 3.1. Isolation, Identification, and Localization of R. electrica in the Digestive Tract of Leafhoppers

We collected adult leafhoppers of *R. dorsalis* to establish a laboratory-rearing system and isolate gut bacteria. Through screening several isolated gut bacteria on an Ashby medium, we were able to obtain a parthenogenic anaerobic strain that exhibited nitrogen fixation activity. The colonies produced translucent, creamy white, round, and convex colonies with a moist and smooth surface when cultured at 28 °C for 2 days on LB agar plates (Figure 1C). Transmission electron microscopy revealed rod-shaped bacterial cells of approximately 3.2 μm in length and 1.1 μm in diameter, with a rod-shaped flagella around the body for swimming and no capsule or spores (Figure 1D).

**Figure 2:**
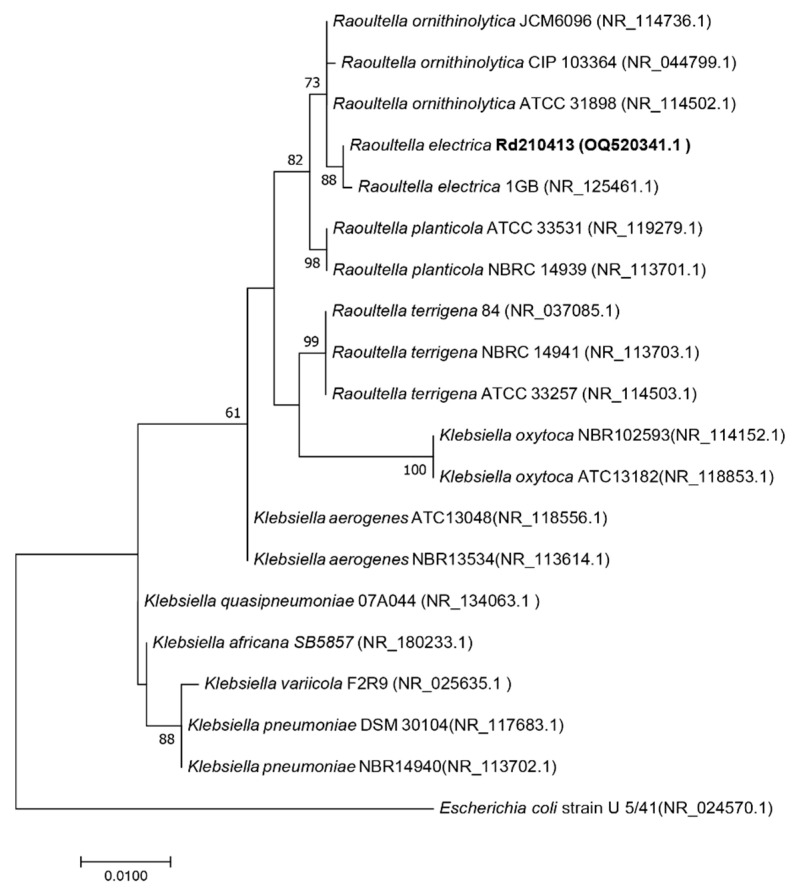
Maximum-likelihood phylogenetic tree based on 16S rRNA gene sequence of *R. electrica* Rd210413 and the type strains of phylogenetically close *Raoultella* species. Bootstrap values (expressed as percentages of 1000 replications) greater than 50% are shown at branch points. The scale indicates the number of amino acid substitutions per site. GenBank accession numbers in parentheses follow the species’ name. *Escherichia coli* strain U 5/41 was used as an outgroup.

**Figure 3:**
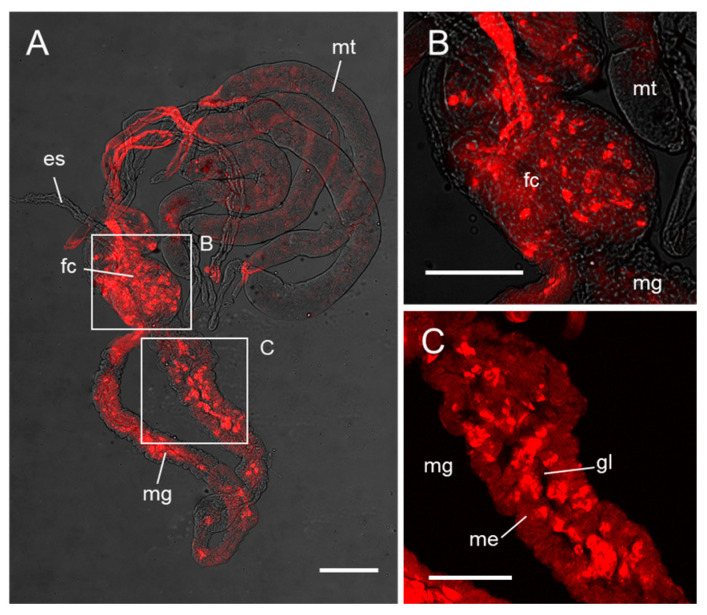
Distribution of *R. electrica* in the digestive tract of the leafhopper *R. dorsalis*. (**A**) Detection of *R. electrica* in the digestive tract of *R. dorsalis* using FISH. (**B**,**C**) *R. electrica* colonizes the filter chamber (**B**) and midgut lumen (**C**) of *R. dorsalis*. (**B**,**C**) are enlargements of the boxed area in (**A**). Scale bars in (**A**): 200 µm; (**B**,**C**): 100 µm. es, esophagus; fc, filter chamber; gl, gut lumen; me, midgut epithelium; mg, midgut; mt, Malpighian tubules.

Through 16S rRNA sequencing, we identified the bacterial strain as *R. electrica* and named it *R. electrica* Rd210413 (GenBank Accession Number OQ595258). Phylogenetic analysis of the 16S rRNA sequence showed that *R. electrica* Rd210413 formed a distinct clade with *R. electrica* strain 1GB within the *Raoultella* genus (Figure 2). We employed fluorescent in situ hybridization with a probe specific to *R. electrica* to investigate its distribution within the digestive tract of the leafhopper *R. dorsalis* (Figure 3). Confocal observations indicated that *R. electrica* was extensively distributed throughout both the midgut and the filter chamber (Figure 3A). Moreover, within the midgut region, *R. electrica* was primarily localized in the lumen of the intestine near the epithelium (Figure 3B,C).

### 3.2. General Genome Features of R. electrica Rd210413

**Figure 4:**
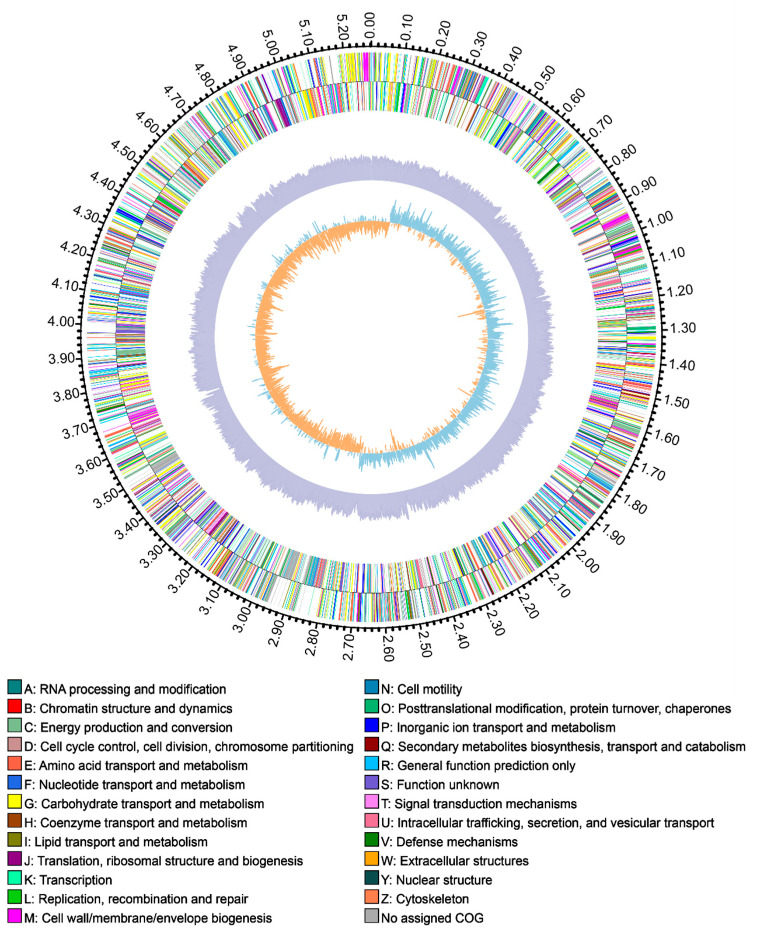
Circular representation of *R. electrica* Rd210413 genome. In the chromosomal DNA map, from the outermost to inner, the circles show nucleotide numbering (circle 1), COG annotation of forward (circle 2) and reverse (circle 3) strands, GC content (circle 4) and GC skew (circle 5).

The genome of *R. electrica* stain Rd210413 was sequenced with a combination of long-read PacBio and short-read Illumina technology. Whole-genome sequencing showed that the *R. electrica* strain Rd210413 only had a single circular chromosome and no plasmid (Figure 4). The general properties of the *R. electrica* genome are presented in Table 1, which comprised 5282709 base pairs of a circular chromosome with an average 55.58% G + C content. It encodes a total of 4890 protein-coding sequences (CDSs), with an average CDS length of 936 bp. The length of the coding regions of the predicted genes accounted for 86.68% of the total genome. In addition, the *R. electrica* genome included 86 tRNA genes, 25 rRNA genes (8 for 16S rRNA, 8 for 23S rRNA, and 9 for 5S rRNA) and 121 sRNA genes were identified in the genome (Figure 4). By aligning the genome to sequences from diverse databases, including the NR, Swiss-Prot, COG, KEGG and GO databases, the numbers of identified genes were 4864, 4207, 4050, 3371, and 3929, respectively.

### 3.3. Nitrogen Metabolism in R. electrica Rd210413

**Figure 5:**
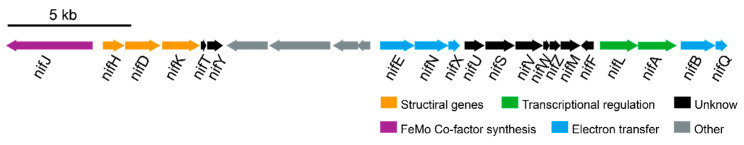
Nitrogen fixation gene clusters of *R. electrica* Rd210413. Color-coded arrows indicate the locations of coding sequences (CDSs) and their orientations.

Based on the genome annotation information, *R. electrica* Rd210413 possesses all the necessary genes for nitrogen fixation, with all these genes located in a 29.4 kbp region of its genome (from nifQ to nifJ) (Figure 5). This region encompasses key components required for nitrogen fixation, including the major structural protein of the nitrogen-fixing enzyme (nifHDK), the transcriptional regulator (nifAL), the FeMo co-factor synthesis gene (nifJ), and the electron transfer gene (nifBENQSUX) (Figure 5).

In addition to its nitrogen fixation capability, *R. electrica* Rd210413 harbors numerous genes related to nitrogen metabolism, implying that it can utilize assimilatory nitrate reduction, dissimilatory nitrate reduction, and denitrification to metabolize nitrogen. This suggests that *R. electrica* Rd210413 can potentially shift between being a source or a sink of nitrogen depending on the growth conditions and can provide nitrogen nutrition to the insects it colonizes.

### 3.4. Phylogenetic Analysis of nifH

**Figure 6:**
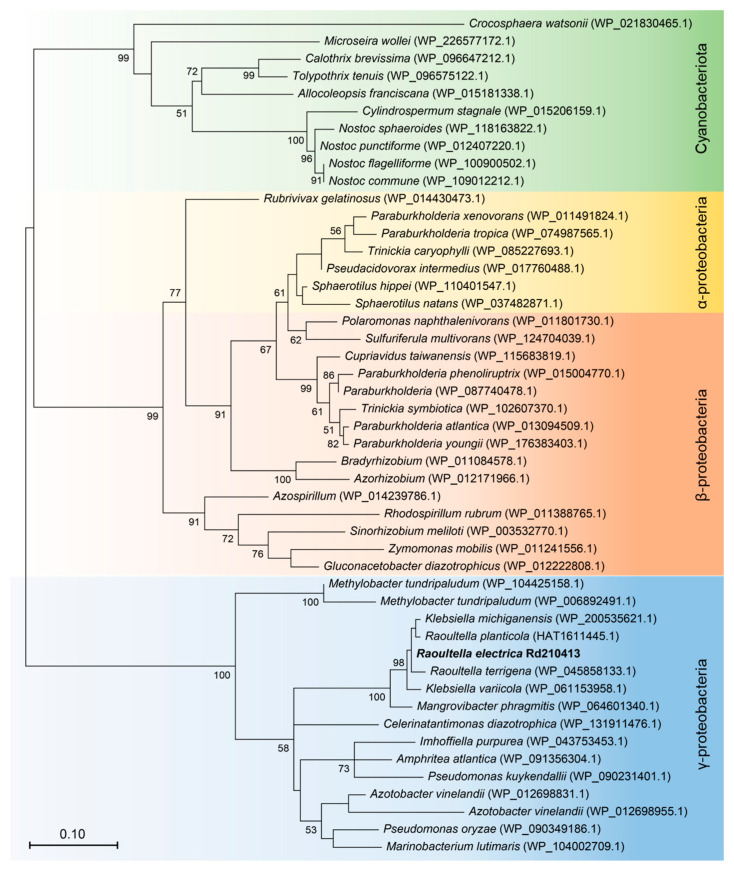
Maximum-likelihood phylogenetic tree of nifH sequences, which include α-, β-, and γ-proteobacterial and cyanobacterial nifH sequences. Bootstrap values (expressed as percentages of 1000 replications) greater than 50% are shown at branch points. The scale indicates the number of amino acid substitutions per site. GenBank accession numbers in parentheses follow the species’ name.

Using the maximum likelihood method, a phylogenetic tree was constructed based on the amino acid sequences of 48 nifH genes to examine the evolutionary relationships between *R. electrica* Rd210413 and other nitrogen-fixing microorganisms (Figure 6). The resulting tree was divided into three distinct clusters, with the first cluster consisting of nifH sequences from cyanobacteria, the second cluster containing nifH genes from α- and β-proteobacteria, and the third cluster containing nifH genes from γ-proteobacteria. *R. electrica* Rd210413 was found in the third cluster and exhibited high similarity with *K. michiganensis*, *R. planticola*, and *R. terrigena* with 98.98%, 98.98%, and 98.63% homology, respectively (Figure 6).

### 3.5. Nitrogen Fixation Capacity of R. electrica Rd210413

**Figure 7:**
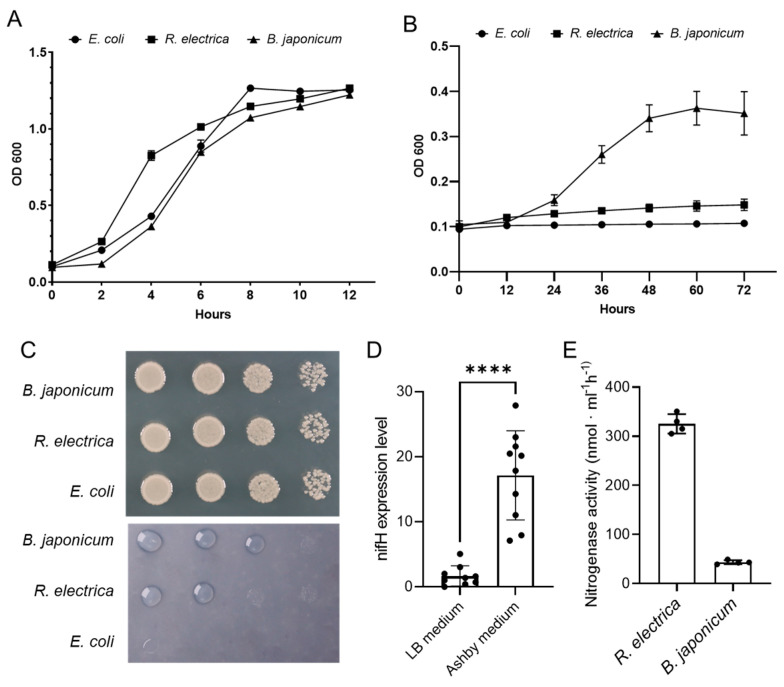
Nitrogen fixation capacity of *R. electrica* Rd210413. (**A**,**B**) Growth curve of of *R. electrica, B. japonicum* and *E. coli* in LB (**A**) or Ashby (**B**) liquid medium. (**C**) Bacterial growth of the *R. electrica, B. japonicum* and *E. coli* in LB (**A**) or Ashby (**B**) agar plate. (**D**) RT-qPCR analysis of nifH gene expression in *R. electrica* grown in LB or Ashby medium. Quantitative data from ten independent experiments are presented as mean ± SD (error bars) values. Significant differences were tested using a *t*-test. **** *p*  < 0.0001. (**E**) Coral-associated nitrogen fixation rates (n = 4 per species) as assessed indirectly from acetylene reduction assays. Nitrogen fixation rates of *R. electrica* were significantly lower than *B. japonicum*. Nitrogen fixation rates are averaged over 24 h (i.e., include light and dark fixation) and presented as mean ± SE. Different letters above bars indicate significant differences between groups (*p* < 0.05).

To assess the nitrogen fixation capability of *R. electrica* Rd210413, we initially compared the growth rates of *R. electrica*, *E. coli*, and *B. japonicum* on LB and Ashby nitrogen-free medium (Figure 7A–C). The results showed that there were no significant differences in the growth rates of the three strains on LB medium, while on Ashby medium, only *R. electrica* and *B. japonicum* were able to grow, with the growth rate of *B. japonicum* being significantly higher than that of *R. electrica*. This suggests that the nitrogen fixation ability of *R. electrica* is inferior to that of *B. japonicum* (Figure 7A–C). In addition, the RT-qPCR results showed that the expression level of the nifH gene in *R. electrica* grown on LB medium was significantly lower than that grown on Ashby medium, indicating that nitrogen-depleted conditions induce the expression of nitrogen fixation genes in *R. electrica* (Figure 7D). In addition, we subsequently evaluated the nitrogen fixation capacity of *R. electrica* using the acetylene reduction activity (ARA) method, which showed a nitrogenase activity of 43.60 nmol·m L^−1^·h^−1^, lower than the nitrogenase activity of *B. japonicum* at 325.34 nmol·m L^−1^·h^−1^ (Figure 7E). The results suggest that *R. electrica* Rd210413 has a low level of nitrogenase activity, which is in line with the predicted function based on the genome analysis.

## 4. Discussion

Leafhoppers, like other herbivorous insects, rely on symbiotic microorganisms for nitrogen nutrition to support their normal growth and development due to imbalances and shortages of nitrogen nutrition in their diet [1,2,3]. Symbiotic microorganisms utilize different metabolic strategies such as nitrogen recycling, fixation, and/or upgrading based on various precursors and physiological requirements [4,5,6,30]. Leafhoppers utilize two primary symbiotic bacteria to provide them with essential amino acids that are absent in their diet and that they cannot synthesize themselves [31]. While the primary symbiotic bacteria of leafhoppers can address the nitrogen nutrition imbalance in their diet by synthesizing essential amino acids, they may not fully meet the overall nitrogen nutrition needs of the leafhopper’s diet [30]. Furthermore, symbiotic microorganisms that can provide nitrogen nutrition through nitrogen recycling or fixation have not been found in leafhoppers. In this research, a novel strain, *R. electrica*, was isolated from the digestive tract of adult leafhopper *R. dorsalis*, which possesses nitrogen fixation ability. We investigated *R. electrica*’s genome and assessed its biological nitrogen-fixing capability. The research data might be explored to understand how gut symbiotic bacteria contribute to insect nitrogen nutrition through biological nitrogen fixation.

Nitrogen is an essential element for the growth and development of all living organisms. Although there is a large amount of nitrogen in the atmosphere, the vast majority of organisms cannot directly use dinitrogen (N2) as a source of nutrients [32]. Nitrogen fixation of symbiotic microorganisms can provide the element requirements for the growth, development, and reproduction of organisms under nitrogen-deficient conditions, as has been widely described in legumes [33]. In insects, symbiotic nitrogen fixation has been extensively studied in termites [6], and it has been demonstrated that nitrogen-containing nutrients synthesized through microbial nitrogen fixation can be assimilated into termite tissues normally [34,35,36]. Direct and indirect evidence of nitrogen fixation has been demonstrated in more than 50 species of termites, but in Hemiptera, only the genera Pentatomidae and Dactylopius have been found to have symbiotic microorganisms for nitrogen fixation [37,38,39,40]. In this study, an *R. electrica* with nitrogen-fixing ability was isolated from the digestive tract of an adult leafhopper *R. dorsalis*.

*R. electrica* is a member of the *Raoultella* genus, which is commonly found in aquatic environments, plants, and soil [17,18,19,20,21]. In recent years, various *Raoultella* spp. bacteria have been discovered with the ability to fix nitrogen, isolated from river water, plant roots, and the intestinal tract of insects [17,18,19]. This study isolated a strain of *R. electrica* bacteria from the adult leafhopper *R. dorsalis*, and FISH experiments revealed that *R. electrica* colonized the filter chamber and midgut of the leafhopper. The nitrogen-fixing ability of microorganisms is determined by the presence of at least six genes coding for structural and biosynthetic components, including nifHDK and nifENB [41]. Genome analysis of *R. electrica* showed that it possesses all the genes necessary for nitrogen fixation, including the major structural protein of the nitrogen-fixing enzyme (nifHDK), the transcriptional regulator (nifAL), the FeMo co-factor synthesis gene (nifJ), and the electron transfer gene (nifBENQSUX). Under conditions of limited nitrogen, exposure to *R. electrica* resulted in a significant upregulation of the critical nitrogen fixation gene nifH, as demonstrated by RT-qPCR analysis. The acetylene reduction assay (ARA) was used to determine the nitrogenase activity [42]. We found that the nitrogen-fixing ability of *R. electrica* was significantly weaker than that of the model nitrogen-fixing bacterium *B. japonicum*. In previous studies, the nitrogen-fixing ability of different strains of nitrogen-fixing bacteria showed significant differences. Generally, the nitrogen-fixing ability of nitrogen-fixing bacteria in plants is significantly higher than that of nitrogen-fixing bacteria from other species. For example, the nitrogen-fixing ability of nitrogen-fixing bacteria from legume root nodules can reach up to 46 μmol acetylene/h/g (dry wt) [43], while the nitrogen-fixing ability of nitrogen-fixing bacteria in stag beetles is only 1.25 nmol acetylene/h/g (fresh wt) [44], which is nearly 1000 times lower.

Moreover, *R. electrica* possesses numerous genes related to nitrogen metabolism, enabling it to convert nitrate to ammonia through assimilatory nitrate reduction, dissimilatory nitrate reduction, denitrification, and more. Ultimately, glutamine can be produced through the action of glutamine synthetase and glutamate synthase and further converted into glutamate. The findings suggest that *R. electrica* may possess the capability to not only perform nitrogen fixation but also potentially utilize other inorganic nitrogen compounds for endogenous amino acid synthesis. In this study, we examined the nitrogen fixation ability of the gut bacterium *R. electrica* and its localization within the digestive tract of *R. dorsalis*. Our findings provide evidence that *R. electrica* is capable of fixing nitrogen and colonizing in the midgut of *R. dorsalis*. However, the question of whether *R. electrica* can provide nitrogen-containing nutrients to its host insect through its own nitrogen fixation ability remains unknown. Further investigations are warranted to elucidate the potential symbiotic relationship between *R. electrica* and its insect host *R. dorsalis*, as well as the putative role of *R. electrica* in facilitating nitrogen nutrient supply for the host insect.

## Figures and Tables

**Table 1:** Genome statistics of *R. electrica* Rd210413.

Features	Chromosome
Genome size (bp)	5,282,709
G + C content (%)	55.58
Protein-coding genes (CDS)	4890
CDS average length (bp)	936
Percent of coding region (%)	86.68
rRNA (5S, 16S, 23S)	9, 8, 8
tRNA	86
sRNA	121
Genes with function prediction	4865

## Data Availability

The genome sequence of *Raoultella electrica* Rd210413 was submitted to NCBI. Corresponding accession numbers with respect to BioProject, BioSample and SRA are as follows PRJNA942203, SAMN33658283 and CP119531. Publicly available data sets were analysed in this study.
